# Reading the Moody chart with a linear interpolation method

**DOI:** 10.1038/s41598-022-10552-x

**Published:** 2022-04-21

**Authors:** Shuxin Huang

**Affiliations:** 1 Department of Engineering Mechanics, Shanghai Jiao Tong University, Shanghai, China; 2 Key Laboratory of Hydrodynamics of the Ministry of Education, Shanghai Jiao Tong University, Shanghai, China

**Keywords:** Engineering, Mechanical engineering, Physics, Fluid dynamics

## Abstract

The error caused by pseudo-midpoint effect in the use of the Moody chart was analyzed. The pseudo-midpoint effect appears in the linear interpolation method usually used to read the Moody chart. The maximum of the error is about 4%, which is significantly lower than the 15% accuracy of the chart in the literature.

## Introduction

The Moody chart provides Darcy friction factors used in the calculations of pipe flows or open-channel flows from the laminar to the turbulent regime^1–3^. The diagram is an essential content of fluid mechanics course for an undergraduate student. All the four textbooks selected and listed in the references^3–6^ contain the Moody chart. An alternative method for obtaining friction factor *f* is to directly adopt the Colebrook equation^1,3,4,6,7^ to calculate *f*, because most of the data in the Moody chart is from the equation. The Colebrook equation is implicit, and an iterative procedure should be employed in the calculation^4,8^. Moreover, many explicit formulas^9–13^ were also proposed for obtaining *f* conveniently.

For a given Reynolds number Re and a relative roughness ε/d, we can read the Moody chart to get *f* without calculation. This is an attractive aspect of the Moody chart especially for the earlier engineering design, e.g. before the 1970s. The advantage of the Moody chart disappears now because we all have a calculator or computer, and we can calculate *f* by using the Colebrook equation or other explicit equations directly. This is also the point of view in Ref.^14^ although Moody chart can also be used in pipe flow directly and easily. However, we have to say that the Moody chart still has an advantage and should be used in teaching because many undergraduate students know little about the algorithms or calculation methods on the Colebrook equation, and because many textbooks, e.g. Refs.^4,6^, only have the Colebrook equation. It is a good mode to read the Moody chart to get *f* at least in teaching.

**Figure 1:**
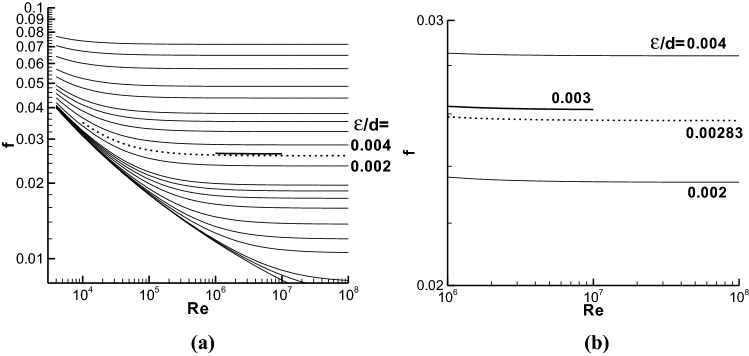
(**a**) A possible error when reading the Moody chart. The relative roughness ε/ds of the lines from the top to the bottom are 0.05, 0.04, 0.03, 0.02, 0.015, 0.01, 0.008, 0.006, 0.004, 0.002, 0.001, 0.0008, 0.0006, 0.0004, 0.0002, 0.0001, 0.00005, 0.00001, 0.000005, and 0.000001 respectively, as used in the Moody chart. The friction factor at ε/d = 0.003 is denoted by bold line, and the dashed line is a pseudo *f* curve at ε/d = 0.003. (**b**) Enlarged figure on the friction factors at ε/d = 0.004, 0.003, 0.00283, and 0.002. The original data is from (**a**).

There could be a problem when using the Moody chart to obtain reliable *f* in the rough regions. For example, Fig. 1a shows the Moody chart but without including the laminar region, and we can see that it is not convenient to get *f* value at ε/d = 0.003. The 0.003 is the midpoint of two adjacent relative roughness ε/d = 0.004 and 0.002 adopted in the Moody chart. Anyone without or with a little experience on the Moody chart usually adopts the dashed line to denote the *f* value at ε/d = 0.003, because the data in the dashed line appears at the midpoint between the *f* values shown at ε/d = 0.004 and 0.002 in the Moody chart. This is a linear interpolation method. However, the relation of *f* to ε/d is a logarithmic function^3–6^ and the logarithmic value of ε/d can not be found easily in the chart. The true *f* value at ε/d = 0.003 is located at the bold line in Fig. 1a, which is slightly higher than the dashed line.

Figure 1b is an enlarged figure of Fig. 1a near ε/d = 0.003. The ε/d = 0.003 is the midpoint between 0.004 and 0.002, but the *f* data in the dashed line at ε/d = 0.00283 could be misread as the *f* at ε/d = 0.003 according to the linear interpolation. This could cause an error in the calculation of pipe flow. The accuracy of the use of the *f* from the Moody chart introduced in the textbook of White^3,12^ is not larger than 15%, and the deviation caused by misusing the data in the dashed line should be identified. The misreading on the *f* value as that at ε/d = 0.003 above is called as pseudo linear interpolation or pseudo interpolation here, and 0.00283 is the pseudo midpoint between ε/d = 0.004 and 0.002. The calculation of 0.00283 can be found in the Method section.

## Results and discussion

### Colebrook equation and Moody chart

The Colebrook equation is as follows^1,3,4,6,7^,

1 $$\frac{1}{\sqrt f } = - 2.0\log \left( {\frac{2.51}{{Re\sqrt f }} + \frac{\varepsilon /d}{{3.7}}} \right).$$

**Figure 2:**
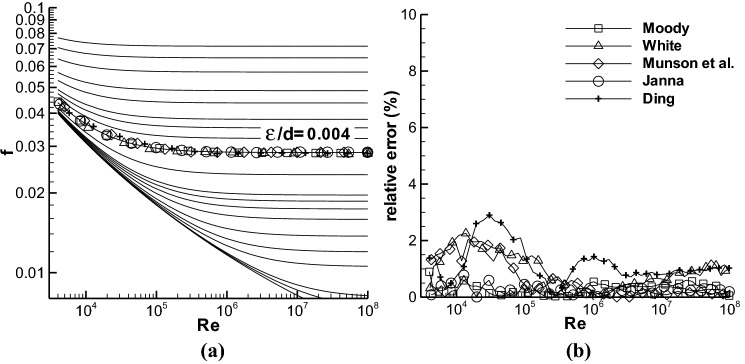
(**a**) The Moody chart in the turbulent region calculated with the Colebrook equation, together with the *f* data at ε/d = 0.004 obtained from four textbooks^3–6^ and the paper of Moody^1^. The ε/ds adopted are the same as those in Fig. 1. The symbols are the data in the references. (**b**) The deviation between the calculated *f* and the data in Refs.^1,3–6^ at ε/d = 0.004. The symbols in (**a**) are the same as in (**b**).

Based on the Re numbers and ε/d values in the Moody chart, the *f* values have been calculated by using Eq. (1) and shown in Figs. 1a and 2a. The *f* data at ε/d = 0.004 read from Refs.^1,3–6^ are also given in Fig. 2a, and the deviations between the data in the references and the present calculation at the roughness are shown in Fig. 2b.

The definition of the deviation is, ${\text{relative}}\;{\text{error}} = \left| {f - f_{{_{{{\text{Colebrook}}}} }} } \right|/f_{{_{{{\text{Colebrook}}}} }} { \times }100,$ where *f* is the data from reference, and *f*_Colebrook_ is the calculation with Eq. (1). The evident error is in the transitional roughness region in Fig. 2b, and the maximum error is lower than 3%, which is similar to the reported maximum error of reading data from the Moody chart in Refs.^11,15^. The results in Fig. 2 indicate that the present calculation of *f* is reasonable.

### Pseudo interpolation error

**Figure 3:**
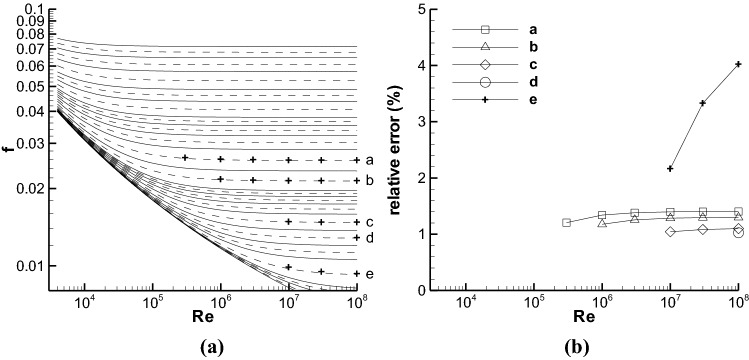
(**a**) The plus symbols denote the locations where the relative errors of the *f*s at the pseudo-midpoint ε/ds in comparison with those at the corresponding midpoint ε/ds are larger than 1%. The nineteen corresponding pseudo-midpoint ε/ds of the dashed lines are 0.0447, 0.0346, 0.0245, 0.0173, 0.0122, 0.0089, 0.0069, 0.0049, 0.0028, 0.0014, 8.94 × 10^−4^, 6.93 × 10^−4^, 4.9 × 10^−4^, 2.83 × 10^−4^, 1.41 × 10^−4^, 7.07 × 10^−5^, 2.24 × 10^−5^, 7.07 × 10^−6^, and 2.24 × 10^−6^ from the top to the bottom. (**b**) The relative errors of the *f*s at five pseudo-midpoint ε/ds, i.e. ε/d = 0.0028, 0.0014, 2.83 × 10^−4^, 1.41 × 10^−4^, and 2.24 × 10^−5^, respectively, from alphabet a–e.

The Moody chart contains twenty-one ε/ds, and the smooth pipe at ε/d = 0 was not calculated and shown in Fig. 1a or 2a. Thus, the twenty ε/ds have nineteen midpoints, which are different from the pseudo midpoints, such as the ε/d of the dashed line in Fig. 1. The misread *f* using the pseudo midpoint of the two *f* curves at any adjacent ε/ds was evaluated by comparing the *f*s at the nineteen pseudo-midpoint ε/ds to the true *f*s at the nineteen midpoint ε/ds at nine Re numbers, i.e. 10^4^, 3 × 10^4^, 10^5^, 3 × 10^5^, 10^6^ 3 × 10^6^, 10^7^, 3 × 10^7^, and 10^8^. Many relative errors between the two kinds of *f*s are lower than 1%. The locations at which the relative errors are larger than 1% are shown in Fig. 3a, and the corresponding relative errors are given in Fig. 3b.

Figure 3b indicates that the maximum error of the misread *f* at the pseudo-midpoint ε/d = 2.24 × 10^−5^ and Re = 10^8^ is about 4%, and most errors is lower than 1.5%. These errors are apparently lower than the 15% inaccuracy of using the Moody chart introduced by White^3^. The influence of pseudo interpolation is not significant. There are two regions that pseudo interpolation could have large relative error. One is near the *f* curve at ε/d = 2.24 × 10^−5^ with large Re, and another is at Re = 10^8^. We can see in Fig. 3b that the error increases with Re, which is also valid for all the other error versus Re curves not shown here. Therefore, the location at Re = 10^8^ should contain the maximum error for the pseudo interpolation phenomenon. However, the *f* at Re = 10^8^ and ε/d = 2.24 × 10^−5^ is seldom used in practice, e.g. in the textbooks of white^3^ and Ding^6^.

**Figure 4:**
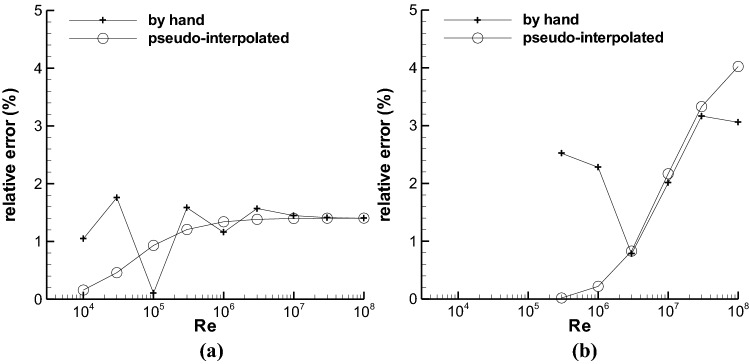
The relative errors obtained by both pseudo interpolation and manual methods, (**a**) at the pseudo-midpoint ε/d = 0.00283, (**b**) at ε/d = 2.24 × 10^−5^.

Figure 4 compares the relative errors of pseudo interpolation to those of manual method, in which the error of manual method is denoted by “by hand”. The manual method means that the *f*s at the two pseudo-midpoint ε/ds, i.e. 0.00283 and 2.24 × 10^−5^, were read directly from the Moody chart in the textbook of Ding^6^ by using a pencil and a ruler. At high Re number, the *f* of by-hand method approaches that of pseudo interpolation in Fig. 4, which indicates that the pseudo interpolation used here is similar to the manual method usually adopted. At the relatively low Re number in the transitional roughness zone, the error of by-hand method is large and approaches to 1–3%, which is similar to the error in Fig. 2b. This indicates that by-hand method could cause 1–3% relative error in the transitional roughness zone.

## Method

### Data

Suppose that two relative roughness ε/ds are 0.002 and 0.004, respectively, as used in Fig. 1b. The pseudo midpoint 0.00283 is calculated as follows,

$$a = \left( {{\text{lg}}\left( {0.00{2}} \right) + {\text{lg}}\left( {0.00{4}} \right)} \right){/2} = \left( {\left( { - {2}.{69897}} \right) + \left( { - {2}.{39794}} \right)} \right)/{2} = - {2}.{54846},$$

and then, the pseudo midpoint is equal to 10^*a*^, i.e., 0.00283.

### Computational method of the Colebrook equation

The bisection method introduced in Refs.^16,17^ was employed to obtain the solution of the Colebrook equation, i.e., Eq. (1).

## Conclusion

The linear interpolation method usually used to read the Moody chart can cause the pseudo-midpoint effect. The present analysis shows that most of the error of friction factor f caused by pseudo midpoint is less than 1.5%, and the maximum of the error is about 4%. The pseudo-midpoint error is much lower than the 15% accuracy of the Moody chart introduced by White^3^. By-hand error is similar to pseudo-midpoint error.

## Supplementary Information

Supplementary Information.

## Data Availability

All data generated or analyzed during this study are included in this published article and its supplementary information file.
